# iDASH secure genome analysis competition 2017

**DOI:** 10.1186/s12920-018-0396-0

**Published:** 2018-10-11

**Authors:** XiaoFeng Wang, Haixu Tang, Shuang Wang, Xiaoqian Jiang, Wenhao Wang, Diyue Bu, Lei Wang, Yicheng Jiang, Chenghong Wang

**Affiliations:** 1 School of Informatics, Computing and Engineering, Indiana University, Bloomington, IN 47408 USA; 2 UCSD Health Department of Biomedical Informatics, University of California San Diego, La Jolla, CA 92093 USA; 3 School of Biomedical Informatics, The University of Texas Health Science Center, Houston, TX 77030 USA

Year 2017 marks the 4th anniversary since the first iDASH Secure Genome Analysis Competition [1] launched jointly by University California San Diego and Indiana University, Bloomington. The past 4 years have witnessed the continued progresses in genomic and biomedical technologies, with their influence permeating our daily life, redefining our perception of privacy. As an example, when the law enforcement identified the golden state killer from his remote relative’s DNA from GEDmatch [2], the kinship inference attack reported 3 years ago [3] now becomes reality. Facing the genome privacy community are still these old devils: protecting the privacy during genomic data sharing and genome analysis. Just they look increasingly real every day, which mounts a great pressure on the young community to come up with practical, usable solutions.

Seeking such practical privacy solutions has always been at the center of the competition, as set by the organizers 4 years ago. This year, serving the purpose are three carefully designed competition tracks, including De-duplication for GA4GH (Track 1), SGX-based whole genome variation search (Track 2) and HME based logistic regression model learning (Track 3). Each track either has its root in the real privacy problem haunting the already deployed system or new challenges emerged from innovative applications of new computing techniques to support secure genomic data sharing or analysis.

Specifically, in collaboration with the Global Alliance for Genomics and Health (GA4GH), Track 1 looks for new privacy-preserving patient linkage (PPRL) solution for removing duplicated health records maintained by multiple data owners. The new solution can be applied on top of existing European ENCCA unified patient identifier framework to facilitate record deduplication in GA4GH. Track 2 is meant to seek new answers for a long-standing genome privacy problem: how to perform a large-scale Genome-Wide Association Study (GWAS) on the untrusted public cloud. This time, however, the attempts are made by leveraging *Intel*’s Software Guard Extension (SGX), a new hardware trusted execution environment (TEE) support, to move a secure analysis solution closer to practical use. Track 3 is designed to answer the new demand for training a machine learning model (logistic regression) on encrypted genomic data, when the computation needs to be conducted in an untrusted environment, through a homomorphic encryption (HME) scheme.

Altogether, these three tracks attracted 65 participation teams from 19 countries across North America, Europe, and Asia. Among them, 19 teams from 23 organizations submitted their final results before the deadline. Finally, a joint team from IBM/INRIA and ENS de Lyon/Cornell Tech/Bar-ILan University won Track 1, CEA France won Track 2 and Seoul National University/UC San Diego won Track 3.

This special issue of *BMC Medical Genomics* highlights some most intriguing techniques reported during the competition.

Carpov et al. [4] describe their winning SGX based secure GWAS solution, which includes two key components i.e., (1) genome data compression and encryption and (2) top K most significant SNPs computation. Rust programming framework was used to enable massively parallel computation, which allows for the application to scale well with large input VCF files. It took about 1 min to handle the whole processing steps for about 30GB inputs.

Laud et al. [5] report the results of using secure multiparty computation to efficiently solve privacy-preserving record linkage problem in large databases. The solution is built upon a commercial platform named Sharemind. To deduplicate 10 million record across 1000 different databases, it took about 30 min for computing servers over 100 Mbits/s network.

Kim et al. [6] present a winning solution for the homomorphic encryption based secure logistic regression. This solution is built upon an efficient approximate arithmetic homomorphic encryption library named HAEEN [7]. The experimental results show that a logistic regression training task over a dataset with 1,579 samples and 18 features can be finished within 6 min.

Chen et al. [8] present another novel solution to the iDASH homomorphic encryption based secure logistic regression task. In their solution, they applied a multi-bit plaintext space in fully homomorphic encryption together with fixed point number encoding. Bootstrapping is combined in fully homomorphic encryption with a scaling operation in the fixed point arithmetics. They also use a minimax polynomial approximation to the sigmoid function and a 1-bit gradient descent method to reduce the plaintext growth in the training process. Their training over encrypted data took 0.4–3.2 h per iteration of the gradient descent.

Bonte et al. [9] discuss an alternative solution for secure logistic regression training over homomorphically encrypted data. The key idea of the proposed solution is based on a simplified fixed Hessian method of a much lower multiplicative complexity, which can be efficiently and iteratively solved under homomorphic operations.

All these new techniques showcase the achievements of this year’s competition. Some of them have already demonstrated the potential of practical use, particularly the deduplication techniques for GA4GH, while the others report exciting progress that can lead to breakthroughs in genome privacy, including truly scalable and privacy-preserving genome analysis on untrusted cloud, based upon the new SGX hardware, and the feasibility of training classification models over fully encrypted data. All such techniques and findings will contribute to the genome-privacy research and move the science in this emerging domain forward.
